# Structured Electrodes Enable High‐Rate and Selective Electrochemical Nicotinamide Adenine Dinucleotide Regeneration for Biocatalysis

**DOI:** 10.1002/cssc.202502221

**Published:** 2026-03-26

**Authors:** Jonas Wolf, Roman Goy, Jonathan Alan Medlock, Julian Tobias Kleinhaus, Kevinjeorjios Pellumbi, Leon Wickert, Daniel Siegmund, Ulf‐Peter Apfel

**Affiliations:** ^1^ Department Power‐To‐Chemicals Fraunhofer Institute for Environmental, Safety and Energy Technology UMSICHT Oberhausen Germany; ^2^ DSM‐Firmenich Kaiseraugst Switzerland; ^3^ Activation of Small Molecules Technical Electrochemistry Ruhr University Bochum Bochum Germany

**Keywords:** biological cofactors, electrochemistry, enzymatic catalysis, hydrogenation, zero‐gap electrolysis

## Abstract

Direct electrochemical regeneration of nicotinamide adenine dinucleotide (NADH) presents a cost‐effective and sustainable alternative to enzymatic recycling approaches, yet its industrial application has been hampered by low reaction rates and insufficient selectivity. In this study, we demonstrate the integration of electrochemical NADH regeneration into a zero‐gap electrolyzer and systematically evaluate copper, silver, and titanium electrodes with respect to activity and selectivity toward 1,4‐NADH. Titanium felt exhibits 100% selectivity for this reaction at a rate of 194 µmol h^−1^ under mild conditions (10 mA cm^−2^). Increasing the current density to 100 mA cm^−2^ significantly enhances the activity, maintaining high selectivity, with titanium and copper electrodes achieving 459 and 258 µmol h^−1^, respectively. Notably, coarser copper meshes further boost 1,4‐NADH formation, reaching 791 µmol h^−1^ at 75% selectivity, underlining the critical role of electrode morphology. This work underscores the potential of scalable, efficient, and selective electrochemical cofactor regeneration, and provides a proof of concept for its application in enzymatic hydrogenation, exemplified by the reduction of acetophenone. We pioneer direct electrochemical NADH regeneration in a zero‐gap electrolyzer, optimizing catalysts for high reaction rates and selectivities. Porous titanium cathodes achieve a selectivity of 100% at reaction rates surpassing the state of the art by a factor of over 3.4. Our findings highlight the scalability of this method for industrial enzymatic catalysis, demonstrated through acetophenone hydrogenation.

## Graphical abstract

Direct electrochemical regeneration of the key enzymatic cofactor NADH is demonstrated in a scalable zero‐gap electrolyzer. Systematic screening of copper, silver, and titanium catalysts reveals that titanium felt achieves 100% selectivity for active 1,4‐NADH, while optimized copper mesh delivers record reaction rates. Electrode structuring and current density are identified as critical handles for balancing rate and selectivity.

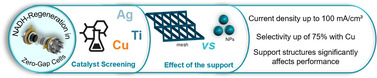

## 1 Introduction

The use of biological cofactors in industrial processes enables unique selectivity in chemical transformations, leveraging the substrate‐specific active sites of catalytically active enzymes [1, 2, 3, 4, 5]. Synthetically used enzymes can either be operated in vivo or outside living organisms, reducing the requirements regarding the toxicity of the reaction environment [6]. A particularly relevant group of enzymes in this regard are oxidoreductases that catalyze valuable reactions, such as the epoxidation of C—C double bonds, oxygenation of C—H bonds, or the reduction of carbonyl groups utilizing nicotinamide adenine dinucleotide (NADH) as a cofactor [1, 7]. However, the high cost of NADH makes its stoichiometric use in large‐scale processes impractical, necessitating methods to regenerate NAD^+^ during enzymatic reactions. Commercial systems predominantly rely on enzymatic regeneration, wherein the enzyme regenerating NADH from NAD^+^ is constantly supplied with fresh co‐substrate [6].

Various strategies have been proposed to incorporate green electricity for NADH regeneration [1]: (i) mediated regeneration, where a redox mediator is reduced on a catalytic surface and subsequently reduces NAD^+^ to NADH [8, 9, 10, 11]; (ii) enzymatic regeneration, in which the mediator aids a reverse enzymatic reaction to regenerate NADH [12, 13, 14]; and (iii) direct reduction of NAD^+^ on a catalytic surface [15, 16, 17]. The inclusion of redox mediators and regenerating enzymes, however, significantly raises costs and presents challenges in terms of stability and separation, which hinder economic feasibility [6, 18].

In contrast, the direct electrochemical reduction of NAD^+^ can reduce system complexity, resource consumption, and overall costs [19]. A significant hurdle in direct NAD^+^ reduction is the formation of inactive derivatives of NADH [20]. The highest reported reaction rate to date is 112 μmol h^−1^ at 6 mA cm^−2^ on a 2.7 cm^2^ copper foam electrode at 78% selectivity for 1,4‐NADH [21], while systems with 100% selectivity operate at much lower rates, with a maximum of 56 μmol h^−1^ at 3 mA cm^−2^ on a 6.3 cm^2^ glassy carbon electrode [15]. The very low reaction rates impair large‐scale applications in coupled enzyme catalysis and cannot be increased unlimitedly by applying higher current densities because of the chemical sensitivity of NADH [6]. The electrode surface area is hence the most critical factor for increasing the overall reaction rate of NADH regeneration. In industrial applications, expanding the electrode area is particularly achievable when using reactor designs that offer high space efficiency and support the assembly of stacks with exchangeable components. Furthermore, to ensure practical scalability, the reactor must maintain high energy efficiency and minimize Ohmic losses during stacking and scale‐up. The zero‐gap electrolyzer is such a reactor type since it features low ohmic losses, inherently good scalability and easily allows stacking of single cells [22].

We herein improve direct NAD^+^ electrolysis using a zero‐gap electroreactor, which provides excellent space efficiency and allows the simple assembly of systems with higher total active surface areas. The latter is a key factor that has limited the large‐scale application of electrochemical synthesis from sensitive organic compounds, such as the direct electrochemical NADH regeneration [22]. Taking advantage of this, we employ an increased active area of 12.6 cm^2^ compared to the state of the art of 6.3 cm^2^ [15] and, with this, seek to increase attainable reaction rate while maintaining high selectivity toward 1,4‐NADH.

## 2 Results and Discussion

### 2.1 Reactor Setup and NADH Quantification

**FIGURE 1:**
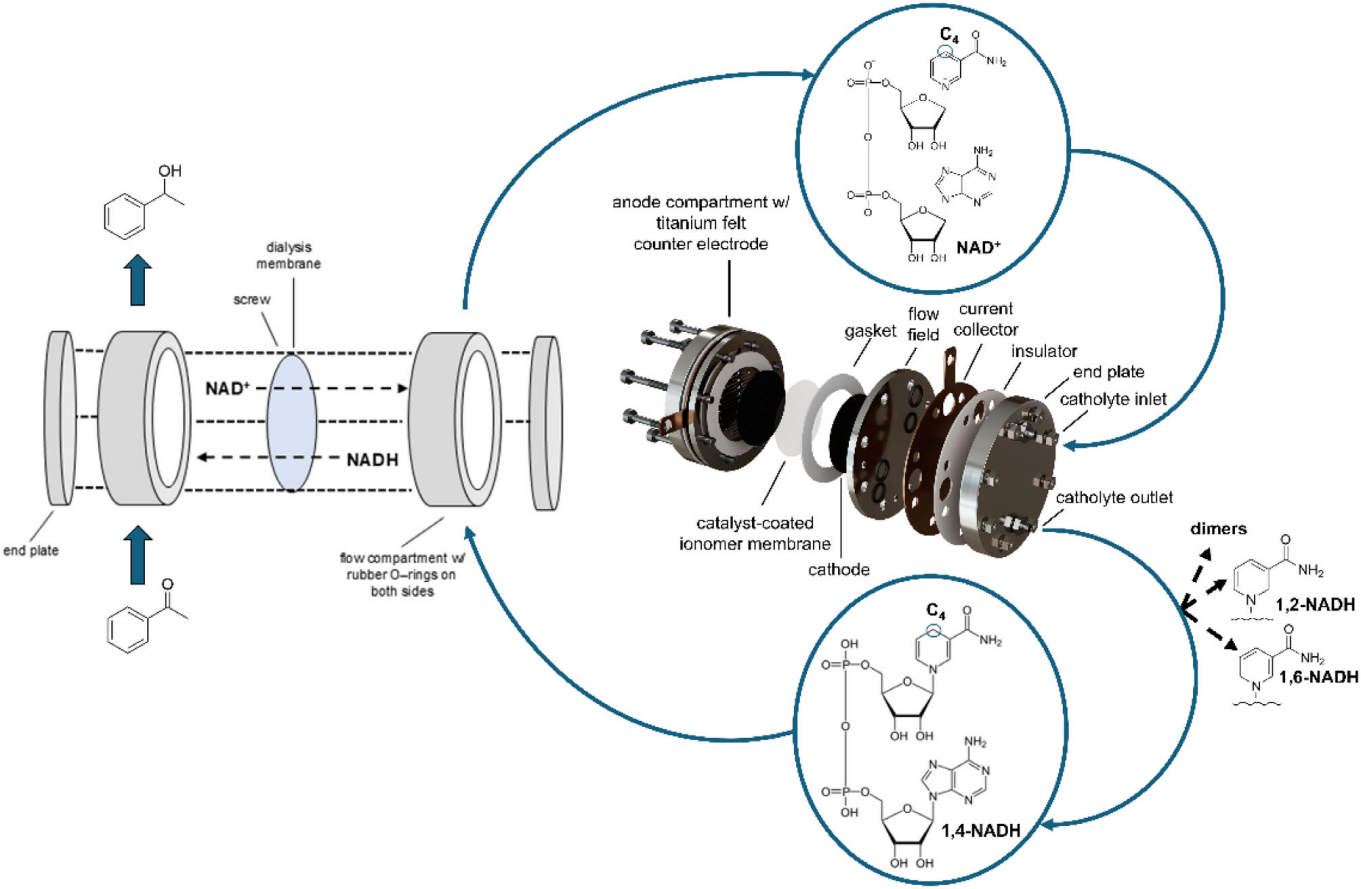
Exploded view of the zero‐gap continuous‐flow electrolyzer used for the direct electrochemical regeneration of reduced nicotinamide adenine dinucleotide (1,4‐NADH) from its oxidized form (NAD^+^), including potential side reactions to isomers and dimers, and its integration with an enzymatic reaction converting acetophenone to 1‐phenylethanol using 1,4‐NADH as a cofactor; the setup illustrates the coupling of electrochemical NADH regeneration with an alcohol dehydrogenase‐catalyzed biotransformation in a two‐compartment flow cell separated by a dialysis membrane. For a detailed photography of the reaction setup, the reader is referred to Figure S4.

Direct electrochemical NADH regeneration was carried out in an in‐house‐made zero‐gap electrolyzer with an active electrode area of 12.6 cm^2^ on three‐dimensional cathodes of varying material, thickness, and porosity (Figure 1). To prevent acidification of the catholyte, we used a tris(hydroxymethyl)aminomethane (Tris)—hydrochloric acid buffer at pH 9, which is known to stabilize NADH, unlike phosphate buffers that can have a destabilizing effect [23]. The conversion was carried out in a semi‐batch continuous flow mode recirculating the electrolyte batches through the reactor. For the counter electrode reaction, we used the oxygen evolution reaction (OER, catalyst: 1 mg cm^−2^ IrO_2_ with electrolyte flow‐rates comparable to the cathodic site) in an acidic medium, an efficient process that avoids limiting the rate of NADH regeneration at the working electrode [24]. The anode and cathode compartments were separated by a Nafion ionomer proton exchange membrane.

In our experiments, the regenerated NADH‐containing electrolyte had an alkaline pH. For quantification of the bioactive 1,4‐NADH, we used a self‐prepared alanine dehydrogenase assay, which converted pyruvate into alanine at pH 9 in the presence of a nitrogen source. The alanine produced was quantified using ^1^H‐NMR spectroscopy (Figure S1). To complement the enzyme assay, UV–vis spectroscopy was used for a broader analysis of the product spectrum, applying the Lambert–Beer law to the absorption at 340 nm (Figure S2) [20]. 1,6‐NADH, 1,4‐NADH, and the dimers of both isomers all absorb at this wavelength so that the reaction rates and Faradaic efficiencies (FE) according to UV–vis spectroscopy correlate with the formation of all these compounds. Hence, in the following, UV–vis‐spectroscopy reflects the overall conversion of NAD^+^ to NADH derivatives and the enzyme assay only that of bioactive 1,4‐NADH, with their ratio being the 1,4‐NADH selectivity [25].

### 2.2 Catalyst Screening

Since factors like mass transport and electrolyte convection around the catalyst layer vary significantly across different reactor designs, catalysts proven in other systems cannot be directly transferred to the zero‐gap configuration [22, 26]. Hence, our initial approach focused on identifying an appropriate catalyst for NADH regeneration in the zero‐gap reactor. Studies have demonstrated that the first single electron transfer (SET) to NAD^+^ typically occurs at a lower half‐cell potential than the second, meaning that at insufficient potentials, the formation of NAD dimers or hydrogenation products, such as 1,6‐NADH or 1,2‐NADH, becomes more favorable than the active 1,4‐NADH [23, 27, 28]. To address these selectivity‐related issues, catalysts must be selected that prevent the excessive favoring of the first SET while promoting hydrogen adsorption [27]. Although several selective direct electrochemical NADH regeneration protocols have been developed, their reaction rates of up to only 112 µmol h^−1^ are currently too low for economic viability. To this end, we chose copper as a catalyst, as several studies have reported its effectiveness in selective direct electrochemical NADH regeneration [21, 29, 30]. We tested copper in different forms, including electroplated porous carbon paper (Cu_EP_), spray‐coated copper nanoparticles on carbon paper (Cu_NP_), and copper mesh (Cu_mesh_). The usage of these different catalyst forms was aimed at providing insights into electrode structuring in the context of process scale‐up. Additionally, titanium in form of porous titanium felt (Ti_felt_) was selected due to its previously reported high selectivity for NADH regeneration, achieving up to 96% selectivity in earlier studies [27, 31], albeit with a relatively low reaction rate below 2 µmol h^−1^. Lastly, we explored the catalytic properties of silver, which demonstrated high efficiency, reaction rate, and selectivity for other electrochemical hydrogenation reactions [32]. Silver was tested in the form of electroplated porous carbon paper (Ag_EP_) and spray‐coated silver nanoparticles on carbon paper (Ag_NP_), similar to the copper configurations. The active area in the case of Cu_EP_ and Ag_EP_ was 7.1 cm^2^ as opposed to 12.6 cm^2^ due to their preparation via electroplating in a 7.1 cm^2^ flow electrolyzer [32]. The catalyst screening was conducted at a current density similar to literature values, namely 10 mA cm^−2^ referring to the geometric electrode area. In comparison, the state‐of‐the‐art reaction rate of 112 µmol h^−1^ at 78% selectivity and of 56 µmol h^−1^ at 100% selectivity were achieved at 6 and 3 mA cm^−2^, respectively [15, 21, 29]. Since one key aim of this work was to investigate the underlying system at increased 1,4‐NADH formation rates, the experiments were carried out galvanostatically. as the current density is most determining for the reaction rate. The reaction rates according to the alanine dehydrogenase assay were determined for selected samples throughout that allowed the highest knowledge gain on the influence of the respective parameter on NADH regeneration.

**FIGURE 2:**
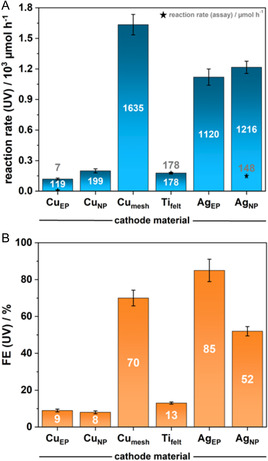
(A) Reaction rate and (B) Faradaic efficiency (FE) of the electrochemical reduction of NAD^+^ according to UV–vis‐spectroscopy and an alanine dehydrogenase enzymatic assay, respectively, upon usage of different configurations of varying metals (EP: electroplated porous carbon paper; NP: nanoparticles spray‐coated on porous carbon paper; v_flow_: 20 ml min^−1^; t_reaction_: 15 min, j: 10 mA cm^−2^).

The screening results revealed that Ag_EP_ and Ag_NP_ exhibited significantly higher FE,85% and 52% for the formation of all NAD derivates, respectively,compared to Ti_felt_ which had an efficiency of 13% and the equivalent copper configurations, which showed 8% and 9% efficiency, respectively (Figure 2, Table 1).

**TABLE 1:** Overview on the results of NAD^+^ electrolysis in a zero‐gap electrolyzer (EP: electrodeposited on carbon paper; NP: nanoparticles spray‐coated onto carbon paper; UV: according to UV–vis spectroscopy; RR: reaction rate; enzyme: according to the alanine dehydrogenase enzyme assay).

Catalyst	**Current density, mA cm** ^**−2**^	**RR (UV), µmol h** ^**−1**^	**RR (enzyme), µmol h** ^**−1**^	Selectivity (1,4‐NADH), %
Cu_mesh_ (fine)	6	97	—	—
Cu_NP_	10	199	—	—
Ag_NP_	10	1216	148	12
Ag_EP_	10	1120	—	—
Cu_EP_	10	119	—	—
Cu_mesh_ (fine)	10	1635	—	—
Cu_mesh_ (fine)	50	7116	—	—
Cu_mesh_ (fine)	75	10 489	148	1
Cu_mesh_ (fine)	100	6708	258	4
Cu_mesh_ (fine)	130	2955	144	5
Cu_mesh_ (fine)^a^	1	650	0	0
Cu_mesh_ (coarse)	100	1438	683	47
Cu_mesh_ (intermediate)	100	1056	792	75
Cu_EP_	30	180	7	4
Cu_EP_ a	30	163	—	—
Cu_mesh_ (fine)	30	597	—	—
Cu_mesh_ (fine)^b^	30	650	—	—
Ti_felt_	100	790	459	58
Ti_felt_	30	356	221	62
Ti_felt_	10	178	194	100
Ti_felt_	130	2570	174	7
Ti_felt_ a	1	471	0	0

a the measurements were carried out in a continuous flow electrolyzer with a membrane‐electrode gap of 1 cm.

b the applied flow rate merely transported a third of the theoretically convertible amount of NAD^+^ through the electrolyzer.

This trend was also reflected in the reaction rates: Ag_NP_ achieved a reaction rate of 1216 µmol h^−1^, the Ti_felt_ 194 µmol h^−1^, and copper nanoparticles 199 µmol h^−1^.

Contrary, Cu_mesh_ with lower active surface area compared to Cu_EP_ and Cu_NP_ outperformed all other metal configurations, reaching a reaction rate of 1635 µmol h^−1^, which challenges the intuitive expectation of higher reaction rates with larger‐surface‐area catalysts. The improved performance of Cu_mesh_ compared to Cu_EP_ and Cu_NP_ may exemplarily stem from better substrate transport to the active sites, especially when compared to carbon‐supported catalysts, where the catalyst layer is situated away from the electrolyte stream.

When it comes to selectivity, Ti_felt_ showed the highest values, consistent with previous literature [27, 31]. This is likely due to its lower affinity for NAD^+^ adsorption, which reduces the likelihood of oligomerization and isomerization. In fact, using the alanine dehydrogenase assay, which selectively measures 1,4‐NADH, we observed a reaction rate of 194 µmol h^−1^ with 100% selectivity compared to 12% for Ag_NP_ and 6% for Cu_EP_. The reaction rate of the 1,4‐NADH formation on Ti_felt_ thus surpasses the state of the art of 56 µmol h^−1^ at 100% selectivity by a factor of over 3.4 [15]. This Ti_felt_ system with 10 mA cm^−2^ operated at a cell voltage of 4.3 V. The zero‐gap electrolyzer likely offers an advantageous environment for the selective and high‐rate formation of 1,4‐NADH compared to previously reported systems. This is primarily due to the strong electrolyte convection near the catalyst layer, which ensures continuous replenishment of the NAD^+^ substrate. This enhanced mass transport likely shortens the average residence time of reactants on the catalyst surface, thereby suppressing the formation of undesired side products.

To gain insights into the potential reasons for the increased reaction rates in the zero‐gap electrolyzer, we compared the NAD^+^ hydrogenation in the zero‐gap electrolyzer on Cu_mesh_ and Ti_felt_ as the most active and most selective catalysts, respectively, with the analogous reaction in a conventional in‐house‐made flow reactor with a membrane‐electrode gap of 1 cm (Figure S3). The flow electrolyzer setup did not allow the application of current densities > 1 mA cm^−2^ at cell voltages < 10 V. This is likely due to the low conductivity of the catholyte, which affects the cell voltage more strongly in the flow electrolyzer compared to the zero‐gap electrolyzer due to the higher membrane‐electrode distance. This underlines the potential of zero‐gap electrolyzers to facilitate the conversion of NAD^+^‐containing electrolytes that do not require exceedingly high supporting electrolyte concentrations, which can interfere with certain downstream enzymatic reactions. The reaction rate according to UV spectroscopy amounted to 650 and 471 µmol h^−1^ for Cu_mesh_ and Ti_felt_, respectively, and no 1,4‐NADH could be detected in the enzyme assay evaluated by ^1^H‐NMR spectroscopy. Reasons for the beneficial outcome in the zero‐gap electrolyzer can be the environment close to the ionomer membrane or the exceedingly high half‐cell potentials favoring side reactions in case of the flow electrolyzer.

To advance understanding and guide future investigations, the application of in situ spectroscopic techniques (e.g., FTIR, Raman) and complementary computational studies (e.g., density functional theory, DFT) is recommended to elucidate the nature of intermediate species and to clarify the influence of electrode morphology on the suppression of side reactions such as dimerization and isomerization.

### 2.3 Optimization of 1,4‐NADH Reaction Rate and Selectivity with Copper and Titanium Catalyst

Comparing the operating costs of the co‐enzymatic regeneration of NADH, which continuously consumes a co‐substrate, with the electrochemical regeneration, the two main competing cost points are the price of the co‐substrate consumed and the electricity price, both per generated equivalent of product,notably under the assumption that the costs for the co‐enzyme can be neglected. Considering sodium formate as an exemplary co‐substrate with a price of 8.96 € kg^−1^ (as a quarter of the price given by small‐scale chemical vendors) [33], the respective costs for the process utilizing enzymatic regeneration amount to 0.61 € mol_product_^−1^ (Table 2).

**TABLE 2:** Cost comparison of processes with enzymatic versus electrochemical NADH regeneration as the enzymatic cofactor (only the costs of the co‐substrate and the consumed electricity were compared, respectively).

Cofactor regeneration route (enzyme – co‐substrate)	**Co‐substrate cost, € kg** ^**−1**^	**Co‐substrate cost, € mol**_**product**_^**−1**^	**Electrochemical electricity cost (idealized** a **), € mol**_**product**_^**−1**^	**Electrochemical electricity cost (demonstrated** b **), € mol**_**product**_^**−1**^	Cost savings versus enzymatic (idealized), %	Cost savings versus enzymatic (demonstrated), %
FDH–sodium formate	8.96 [33]	0.61	0.02	0.164	97	73
GDH–glucose	3.73 [33]	0.25	0.02	0.164	92	34

a Idealized assumes 100% FE and 100% 1,4‐selectivity. Electricity price = 0.09 € kWh^−1^.

b Demonstrated: FE = 21%, 1,4‐selectivity = 58% (100 mA cm^−2^).

Assuming an electricity price of 0.09 € kWh^−1^ [34], a cell voltage of 4.3 V, a current density of 10 mA cm^−2^, and an electrode area of 12.6 cm^2^ (which are a set of parameters employed in this study), the respective costs for the process utilizing direct electrochemical regeneration amount to 0.02 € mol_product_^−1^. This signifies cost savings of 97% for the process utilizing electrochemical, as opposed to enzymatic, regeneration of the cofactor. Considering glucose–glucose dehydrogenase as an enzymatic NADH‐regenerating system, the respective costs and the cost savings would amount to 0.25 € mol_product_^−1^ and 92%, respectively, when assuming a glucose price of 3.73 € kg^−1^ (as an approximate quarter of the price given by small‐scale chemical vendors) [33]. However, these results are only valid when assuming a Faradaic efficiency and selectivity of the electrochemical regeneration of 100%. High selectivities are hence crucial for an economical operation of continuous enzymatic catalysis due to the high prices for NAD^+^ [35]. Beyond the idealized upper‐bound benchmark (100% FE/selectivity), we additionally considered a demonstrated‐performance scenario of the high current density regime (100 mA cm^−2^, FE = 21%, Table S1), which increases the electricity‐normalized NADH cost by ≈4.8× under otherwise identical assumptions (0.095 € mol_product_^−1^). Furthermore, accounting for the experimentally demonstrated 1,4‐selectivity (58%, Table S1) results in 0.164 € mol^−1^ for bioactive 1,4‐NADH under otherwise identical assumptions.

**FIGURE 3:**
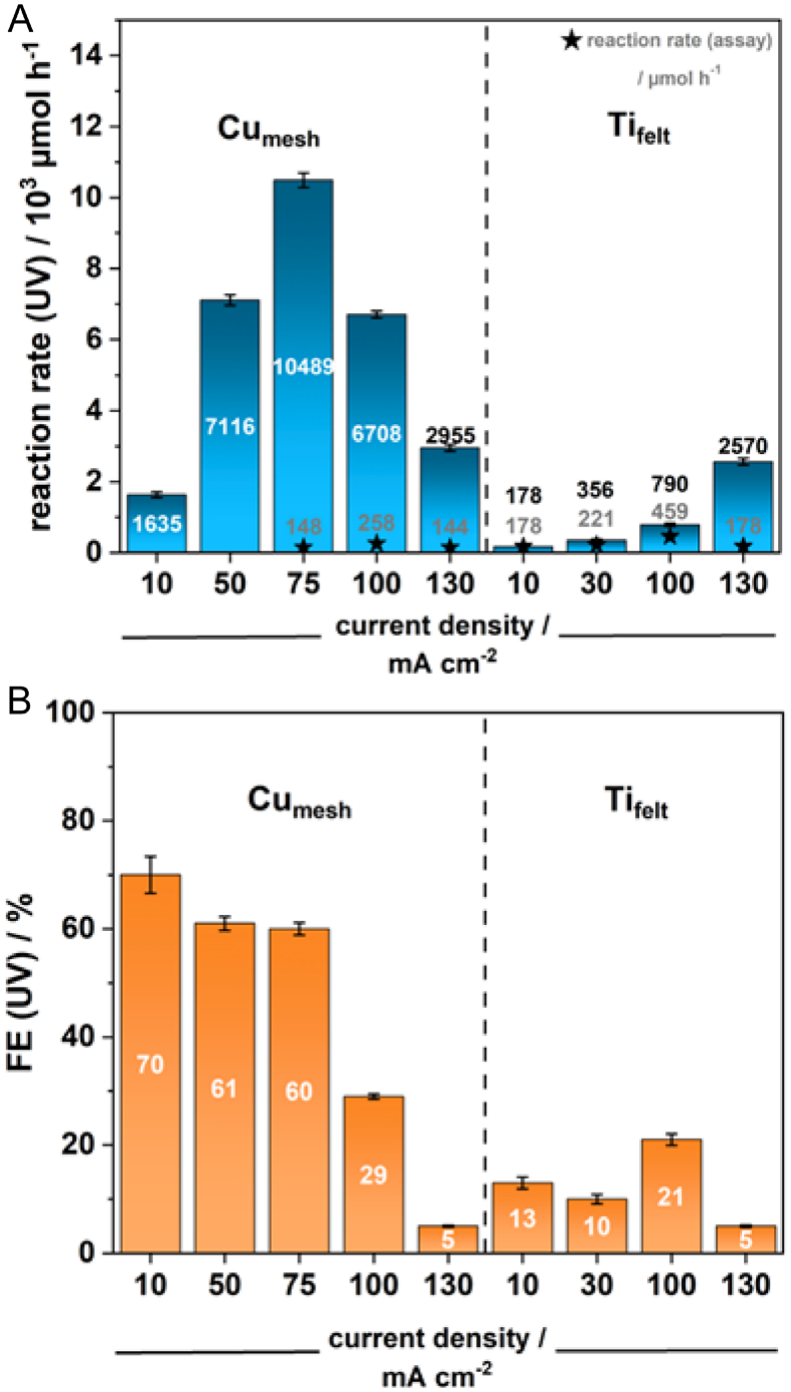
(A) Reaction rate and (B) Faradaic efficiency (FE) of the electrochemical reduction of NAD^+^ on copper mesh and titanium catalysts according to UV–vis‐spectroscopy and an alanine dehydrogenase enzymatic assay, respectively, upon application of varying current densities (the reaction rate according to the enzyme assay for Ti_felt_ at a current density of 10 mA cm^−2^ is given as 178 µmol h^−1^ corresponding to a selectivity of 100%, cathode: Cu_mesh_; v_flow_: 20 ml min^−1^; t_reaction_: 15 min).

Given that copper exhibited the highest reaction rate for NADH‐derivative formation, our goal was to optimize the reaction conditions to maximize 1,4‐NADH selectivity. Ti_felt_ showed the best selectivity for 1,4‐NADH of 100% in the herein‐presented experiments so that the focus of optimization with respect to Ti_felt_ was the reaction rate. Current density significantly impacts the electrode half‐cell potential, which in turn influences substrate adsorption and desorption during electrosynthesis and can cause either of the two previously mentioned SET steps to prevail. Thus, we hypothesized it would impact the selectivity for 1,4‐NADH. As such, we tested current densities of 10, 50, 75, and 100 mA cm^−2^, using Cu_mesh_ and Ti_felt_ as the cathode materials (Figure 3, Table 1).

Notably, the highest overall rate of NADH derivative formation did not correspond to the highest yield of 1,4‐NADH. At 75 mA cm^−2^, UV spectroscopy indicated a formation rate of 10.5 mmol h^−1^, while the enzyme assay showed 148 µmol h^−1^, resulting in a low selectivity of just over 1%. At 100 mA cm^−2^, the rates were 6.7 mmol h^−1^ (UV) and 258 µmol h^−1^ (enzyme assay), with a selectivity of 4%. Moreover, the increased selectivity at higher current densities for copper is consistent with literature findings, which show that higher half‐cell potentials, naturally promoted by higher current densities, lead to enhanced 1,4‐NADH selectivity [28]. Enhancing the current density even further to 130 mA cm^−2^, however, decreases the reaction rate both toward 1,4‐NADH (enzyme assay) to 144 µmol h^−1^ and to other NADH isomers (UV) to 3.0 mmol h^−1^. Potentially, a given threshold half‐cell potential is surpassed at this point, beyond which decomposition of NAD^+^ to unknown side products is favored.

For the Ti_felt_ catalyst, the FE and reaction rate of the total amount of NADH derivates as well as the 1,4‐NADH formation rate increase until 100 mA cm^−2^. The best 1,4‐NADH formation rate obtained for Ti_felt_ amounts to a notable 459 µmol h^−1^. The increasing FE at higher current densities suggests that mass transport limitations were not a factor in this case. This reaction rate represents the highest for direct electrochemical NADH regeneration reported to date. A current density of 130 mA cm^−2^ results in a decrease of the 1,4‐NADH reaction rate to 178 µmol h^−1^ and of its selectivity to 7%. A maximum 1,4‐NADH reaction rate can thus be expected between 100 and 130 mA cm^−2^. Hence, the trend of increasing selectivities with enhanced current densities is not applicable to Ti_felt_, which likely features an inherently favorable half‐cell potential for the first SET to NAD^+^ even at low current densities. When a given threshold half‐cell potential is surpassed, substrate adsorption is potentially facilitated, and the resulting higher substrate coverage of the catalyst layer makes isomerization and dimerization take over.

### 2.4 Variation of Copper Mesh Morphology

**FIGURE 4:**
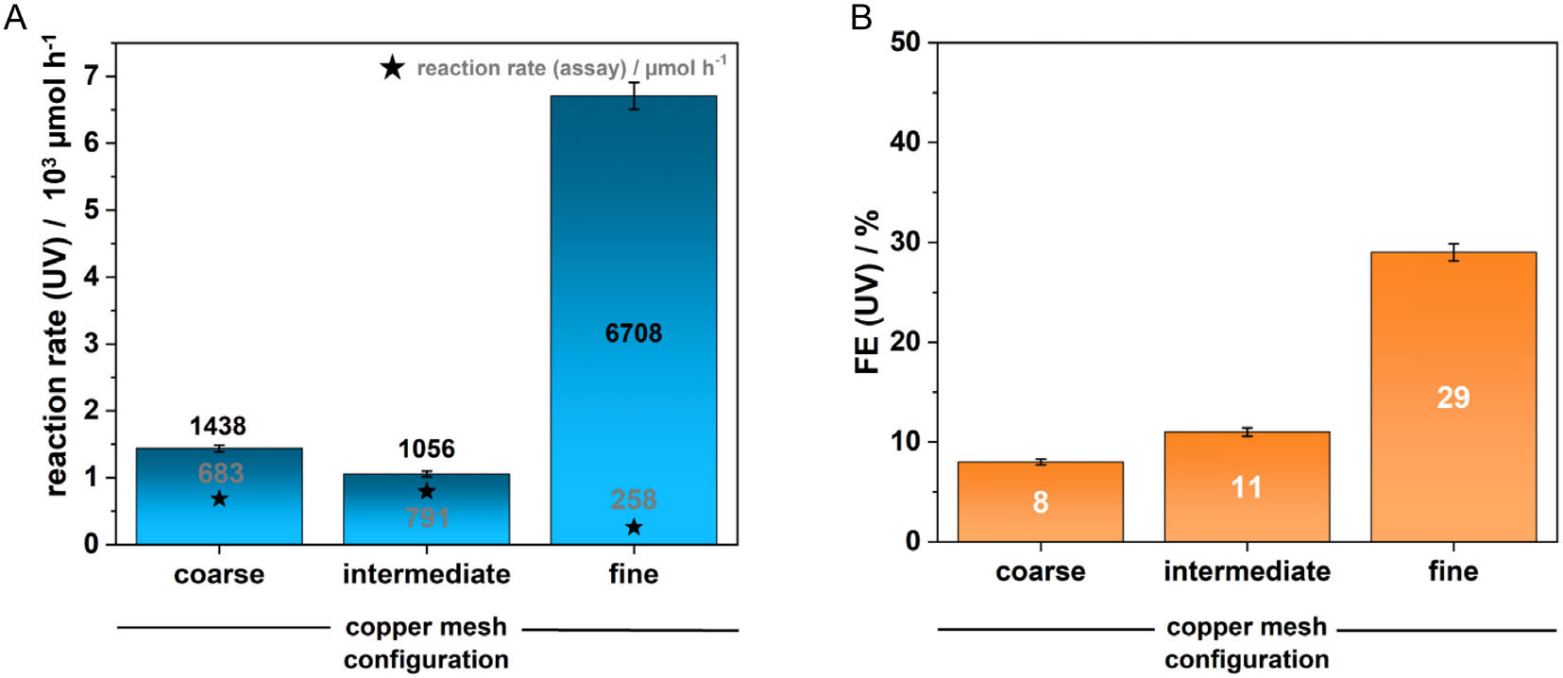
(A) Reaction rate and (B) Faradaic efficiency (FE) of the electrochemical reduction of NAD^+^ on copper mesh with varying mesh sizes according to UV–vis‐spectroscopy and an alanine dehydrogenase enzymatic assay, respectively (coarse: 10 apertures per inch, 0.15 mm wire diameter; intermediate: 50 apertures per inch, 0.18 mm wire diameter; fine: 140 apertures per inch, 0.056 mm wire diameter; thickness of all Cu_mesh_: 0.3 mm; j: 100 mA cm^−2^; v_flow_: 20 ml min^−1^; t_reaction_: 15 min).

To investigate the influence of active surface area and convection dynamics on NADH regeneration, we tested several types of Cu_mesh_: a coarse one (10 apertures per inch, 0.15 mm wire diameter), intermediate one (the previously used Cu_mesh_ with 50 apertures per inch and 0.18 mm wire diameter), and a fine option (140 apertures per inch, 0.056 mm wire diameter). Electrolysis was conducted at a current density of 100 mA cm^−2^, which had previously shown the best selectivity for 1,4‐NADH (Figure 4, Table 1).

The finest Cu_mesh_ demonstrated the highest FE of 29% and the greatest reaction rate of 6.7 mmol h^−1^, according to UV–vis spectroscopy, indicating the highest conversion of NAD^+^ to NADH derivatives. This could be attributed to the increased availability of active sites, though this also led to a higher rate of side reactions. In contrast, the coarser Cu_mesh_, while showing lower NAD^+^ conversion rates and FEs, exhibited significantly higher 1,4‐NADH formation rates of 683 µmol h^−1^ for the coarsest mesh and 791 µmol h^−1^ for the intermediate mesh. These rates surpassed the state‐of‐the‐art results obtained with copper foam of 112 µmol h^−1^ with respect to the total 1,4‐NADH formation rate and also the rate per unit area that amounts to 63 µmol h^−1^cm^−2^ in this study and to a maximum of 42 µmol h^−1^cm^−2^ in literature [21]. The obtained values even exceed the performance of the Ti_felt_ catalyst used in this study albeit, at lower selectivity. These findings underscore the importance of macroscopic electrode structuring for specific process parameters and provide a solid starting point for optimized enzyme catalysis.

### 2.5 Coupling NADH Regeneration to Enzyme Catalysis

To provide a proof of concept for coupling the electrochemical NADH regeneration with enzyme catalysis, we set up the above‐described electrochemical zero‐gap cell and led the respective catholyte to a chamber that separated the catholyte from an enzyme solution by a dialysis membrane with a molecular weight cut‐off (MWCO) of 3.5 kDa to allow NADH and substrate to diffuse freely with the enzyme being contained in its compartment. The enzyme solution was led to another third reservoir next to catholyte and anolyte. The catholyte in this case was identical with the one used in the rest of this study, except for 6.5 g L^−1^ of acetophenone being added. It was chosen as the model substrate due to its relevance in the production of pharmaceuticals, resins, and fragrances [36] and to thus prove the applicability of the directly electrochemically generated NADH stream to relevant enzymatic reactions. The enzyme solution, additionally to acetophenone, contained 1 g L^−1^ bovine serum albumin and 0.3 g L^−1^ of a specialized alcohol dehydrogenase (Figures S4, S5). During operation of the setup at 10 mA cm^−2^ with a Ti_felt_ cathode for 24 h, a reaction rate for the acetophenone conversion to 1‐phenylethanol of 33 µmol h^−1^, determined by ^1^H‐NMR spectroscopy, was obtained (Table 3).

**TABLE 3:** Formation rates of 1‐phenylethanol from acetophenone catalyzed by a model alcohol dehydrogenase depending on different parameters; the enzyme solution was separated from the catholyte of an electrochemical reactor by a dialysis membrane through which it was supplied with NADH formed by electrochemical regeneration of NAD^+^ (MWCO: molecular weight cut‐off).

Number	Enzyme	Dialysis membrane (MWCO in kDa)	**1‐Phenylethanol formation rate, µmol h** ^**−1**^
1	ADH_105	3.5	33
3	ADH_27 (26% as active as ADH_105)	3.5	50
4	ADH_105	6–8	80

The lower reaction rate compared to that of the neat NADH regeneration at these conditions of 194 µmol h^−1^ (with one equivalent of acetophenone indirectly consuming one equivalent of NADH)may originate from mass transport limitations through the dialysis membrane since enzymatic reactions would usually be fast enough to process the largest share of NADH formed over 24 h [37]. To verify this assumption, we ran a control experiment with a compartment containing NADH in 1:1 stoichiometric ratio to the present acetophenone rather than the electrochemical regeneration reactor. Merely 0.2% of the present NADH was consumed after 24 h, which leaves mass transport limitation by the dialysis membrane or the inherent enzyme activity as options for the bottle neck. A second control experiment was carried out in which another alcohol dehydrogenase—with an inherent activity of only 26% of that of the previously employed enzyme—was used. An acetophenone conversion rate of 50 µmol h^−1^ resulted, which, compared to 33 µmol h^−1^ obtained with the more active enzyme, confirms the mass transport through the dialysis membrane as the bottle neck for acetophenone conversion in the coupled reactor. Employing the original setup, but rather with a dialysis membrane with a MWCO of 6–8 kDa, increased the 1‐phenylethanol formation rate to 80 µmol h^−1^ which yet again confirms the membrane as the bottle neck. Since this rate still does not approach the potential of the NADH regeneration of 194 µmol h^−1^ and an even higher MWCO could result in enzyme crossover, alternative separation concepts of electrochemical and enzymatic compartment must be considered in the future. At constant current density and maintained selectivity, the 1,4‐NADH production rate is expected to scale approximately linearly with the aggregated cathode area. Key technical open issues for scale‐up are here maintaining 1,4‐selectivity at higher throughput/residence‐time distributions and implementing an enzyme‐electrolyzer integration that avoids enzyme exposure to electrode potentials while ensuring cofactor‐accessability. Herein, the enzyme could be immobilized within the reactor, however not in direct contact to the electrode surface to protect it from potential decomposition, or the NADH as the cofactor could be immobilized on the electrode surface, preventing access of the enzyme to the electrode and avoiding mass transport problems [38]. Concludingly, the above experiments demonstrate the applicability of the herein‐presented protocol of NADH regeneration in enzyme catalysis, rendering further investigations promising. Conceptually, the presented platform should be transferable to NADP^+^/NADPH regeneration, as the underlying electrochemical driving force and reactor scaling principles remain similar. However, the additional 2′‐phosphate group in NADP^+^ can alter interfacial interactions, transport, and potentially the selectivity between 1,4‐ and other reduced isomers, which may require re‐optimization of electrode material, operating window, and electrolyte composition. In addition, NADPH‐dependent biocatalytic modules differ significantly, so that compatibility of the electrochemical output must be validated in future studies.

## 3 Conclusion

This study aimed to implement the direct electrochemical regeneration of NADH into a scalable zero‐gap electrolyzer. We utilized UV–vis spectroscopy to reflect NAD^+^ conversion to NADH derivates and an alanine dehydrogenase enzymatic assay to separately quantify 1,4‐NADH production. A comparative analysis of copper, silver, and titanium catalysts in various configurations revealed that the copper mesh catalyst had the highest overall NAD^+^ conversion rate to NADH derivatives of 10.5 mmol h^−1^, while the titanium felt catalyst achieved the highest selectivity for 1,4‐NADH (100%) at a rate of 178 µmol h^−1^. The zero‐gap electrolyzer likely enables faster and more selective 1,4‐NADH production compared to state‐of‐the‐art systems by enhancing electrolyte convection near the catalyst, which improves NAD^+^ supply, reduces its average dwell time and reduces side reactions. Increasing the applied current density from 10  to 100 mA cm^−2^ significantly enhanced the 1,4‐NADH formation rate, with copper and titanium achieving rates of 258 and 459 µmol h^−1^, respectively, while also improving the selectivity for copper compared to lower current densities. Interestingly, using coarser copper meshes led to a 1,4‐NADH formation rate of 791 µmol h^−1^, with a selectivity of 75%, potentially due to the lower active area, which helped minimize side reactions. These findings highlight the critical role of macroscopic electrode structuring in optimizing NADH regeneration within the zero‐gap electrolyzer setup. This study reports a high reaction rate for direct electrochemical NADH regeneration with good selectivity using copper and excellent selectivity using titanium both in the form of commercially available electrode materials. A proof of concept for the coupling of this direct electrochemical NADH regeneration method with enzyme catalysis is provided using acetophenone hydrogenation as an example. This work hence lays the groundwork for the transfer of enzyme catalysis to scalable, stackable, and thus more economically viable electrochemical reactor systems.

## Supporting Information

Additional supporting information can be found online in the Supporting Information section. **Supporting** **Fig.** **S1:** Exemplary 1H‐NMR spectrum of a product sample after conduction of the alanine dehydrogenase assay and the corresponding enzymatic reaction scheme (the marked doublet corresponds to the methyl group of alanine; its integral compared to that of an internal standard was used to determine the amount of present 1,4‐NADH). **Supporting** **Fig.** **S2:** UV‐Vis spectrum of an exemplary product sample from the direct electrochemical reduction of NAD^+^ (absorption band at 260 nm corresponds to all NADH derivates; absorption band at 340 nm corresponds to 1,4‐NADH and potentially to 1,6‐NADH and the oligomers of both isomers). **Supporting** **Fig.** **S3:** Continuous flow reactor configuration used for the direct electrochemical regeneration of NADH from NAD+ (the electrolytes were recirculated through the cell using a peristaltic piston pump). **Supporting** **Fig.** **S4:** Image of the enzymatic conversion of acetophenone to 1‐phenylethanol with an NADH‐dependent alcohol dehydrogenase coupled with the electrochemical regeneration of NADH. **Supporting** **Fig.** **S5:** ^1^H‐NMR‐spectrum of the enzyme solution after operation of the setup from Figure S4 for 24 h at a current density of 10 mA cm^‐2^. **Supporting** **Table S1:** Overview of the electrolysis experiments of NAD^+^ in a zero‐gap electrolyzer (EP: electrodeposited on carbon paper; NP: nanoparticles spray‐coated onto carbon paper; UV: according to UV‐Vis spectroscopy; RR: reaction rate; enzyme: according to the alanine dehydrogenase enzyme assay).

## Funding

This work was supported by Studienstiftung des Deutschen Volkes, Deutsche Forschungsgemeinschaft (AP242/22‐1, AP242/23‐1 and EXC2033‐390677874‐RESOLV ), Bundesministerium für Bildung und Forschung (031B1403A).

## Conflicts of Interest

The authors declare no conflicts of interest.

## Supporting information

Supplementary Material

## Data Availability

The data that support the findings of this study are available from the corresponding author upon reasonable request.
