# Supplementary material for: Structured Electrodes Enable High‐Rate and Selective Electrochemical Nicotinamide Adenine Dinucleotide Regeneration for Biocatalysis
Source: ChemSusChem. 2026 Mar 26;19(6):e202502221. doi: 10.1002/cssc.202502221 (PMC13021300; doi:10.1002/cssc.202502221)
Supplement: Supplementary file 1 — Supplementary Material [file CSSC-19-e202502221-s001.pdf]

Structured Electrodes Enable High-Rate and Selective Electrochemical NADH Regeneration for Biocatalysis

Jonas Wolf[a], R. Goy[b], Jonathan Alan Medlock[b], Julian Tobias Kleinhaus[c], Kevinjeorjios Pellumbi[a], Leon Wickert[c], Daniel Siegmund[a,c]*, Ulf-Peter Apfel[a,c]*

[a] J. Wolf, Dr. K. Pellumbi, Dr. D. Siegmund, Prof. Dr. U.-P. Apfel
Department of Electrosynthesis
Fraunhofer Institute for Environmental, Safety and Energy Technology
Osterfelder Straße 3, 46047 Oberhausen, Germany
E-mail: ulf.apfel@ruhr-uni-bochum.de, daniel.siegmund@umsicht.fraunhofer.de

[b] Dr. R. Goy, Dr. J. Medlock
DSM-Firmenich
Wurmisweg 576, 4303 Kaiseraugst, Switzerland

[c] Dr. J. T. Kleinhaus, L. Wickert, Dr. D. Siegmund, Prof. Dr. U.-P. Apfel
Activation of Small Molecules, Technical Electrochemistry
Ruhr University Bochum
Universitätsstraße 150, 44801 Bochum, Germany

Table of Contents

Materials 1

Cell setup and measurement protocol 1

Analytic methods 2

Analytic spectra for the quantification of NADH 3

Flow cell setup 4

Setup coupling electrochemical NADH regeneration with enzyme catalysis 5

Compiled data of all conducted measurements 6

Materials

All chemicals were utilized without further purification. β-NAD^+ hydrate, tris(hydroxymethyl)aminomethane and ammonium chloride were sourced from *Fisher Scientific*. Hydrochloric acid, potassium hydroxide, alanine dehydrogenase (recombinant; >15 units mg^{-1}), sodium pyruvate, silver nanoparticles (particle size <100 nm), copper nanoparticles (particle size: 25 µm), and sulfuric acid were obtained from *Sigma-Aldrich*. Silver nitrate was purchased from *Carl Roth*. NADH disodium salt hydrate (NADH) was purchased from *Sigma-Aldrich* and bovine serum albumin (BSA) from *Carl Roth*. The alcohol dehydrogenase ADH-105 was bought from *Johnson Matthey*. The carbon paper was H23/E20 (thickness: 210 µm, without MPL) supplied by *Freudenberg*. All copper meshes (10, 50, 140 apertures per inch, wire diameters: 0.15, 0.18, 0.06 mm respectively, thickness 0.3 mm) as well as the 3,500 Da MWCO SnakeSkin dialysis tubing were supplied by *Fisher Scientific*. The 6,000-8,000 Da MWCO dialysis disc membrane was purchased from *Repligen*. The respective flow cell for coupling enzyme catalysis and NADH regeneration was built in-house using polyether ether ketone. Nafion 115 membrane from *Chemours* was supplied by *Quintech*. Titanium felt, 0.15 mm thick, was sourced from *Bekaert Fibre Technologies*.

Cell setup and measurement protocol

The electrochemical regeneration of NAD^+ took place in a zero-gap electrolyzer, as detailed in literature including its periphery.[20,26,29–32] In short, the house built single cell electrolyzer features an electrode area of 12.57 cm^2 (40 mm diameter). The anode was run in CCM mode as described below. The cell is sealed using PTFE gaskets and tightened using a torque value of 5 N m over eight screws to ensure a leak-less operation. Ti-flow-fields with a serpentine structure ensure optimal distribution of the reactant solutions/electrolytes. A copper plate in direct contact with the flow fields served as the current collector plate. The iridium oxide catalyst was spray-coated onto the anode side of the Nafion 115 membrane according to an ink formulation and method described in literature.[29] 0.15 mm-thick titanium felt served as the anode's porous transport layer, with 10 mM H_2SO_4 as the anolyte. The catholyte volume was optimized for theoretical full conversion at the respective transported charge (**Table S1**). The electrode active area was 7.1 cm^2 for the electrodeposited electrodes and 12.6 cm^2 for all other configurations. Electrodeposited electrodes were prepared following a procedure from literature.[26] Nanoparticle-based electrodes were spray-coated following an ink formulation and coating techniques outlined in literature.[26] The NAD^+ concentration in the catholyte was 8 g l^{-1} and the duration of electrolysis was 15 min throughout. The electrolyte volume was chosen so that the theoretically convertible amount of NAD^+ was present (**Table S1**).

Where denoted, electrolysis was carried out in a continuous flow reactor with a membrane-electrode gap of 1 cm (**Figure S3**). It consisted of two 1 cm-thick, in-house made polyether ether ketone (PEEK) flow compartments that separated the electrodes from the membrane. Tightness of the cell was ensured by fluorocarbon rubber O-rings and gaskets surrounding the electrodes and the membrane. The electrodes were electrically contacted directly with copper plates. The copper plates were insulated towards PEEK end plates with fluorocarbon rubber gaskets.

Analytic methods

For analysis, UV-Vis spectroscopy was performed using a *Tecan* Infinite 200 Pro plate reader with 96-well plates. A 50 µL sample volume was used, calibrated with six NADH standards ranging from 0.3 mM to 1.5 mM. Additional analysis was conducted using an alanine dehydrogenase assay. The procedure involved adding 10 mg of BSA, 4 mg of sodium pyruvate, and 26 mg of ammonium chloride to 10 mL of the respective NADH-containing electrolyte, followed by degassing with argon. Five units of alanine dehydrogenase were added, and the mixture was stirred at 37 °C for 3 hours. A reference solution consisting of 20 mg neat NADH in 50 mM Tris-buffer (pH 9) was used. The alanine formed was quantified using ^{1}H-NMR spectroscopy on a *Bruker Avance Neo 400* with potassium dihydrogen phthalate serving as the internal standard (**Figure S1**). The determination of the relative activity of ADH_105 and ADH_27 was done with an enzyme assay analogous the previously mentioned reference solution, however with acetophenone as the substrate. The content of 1-phenylethanol as the reaction product was then quantified *via* ^{1}H-NMR spectroscopy. The FE according to UV-Vis spectroscopy signifying the FE for the formation of all NADH derivates was calculated assuming a consumption of two electrons per molecule of NAD^+.

Before delving into the implementation of NADH regeneration into a zero-gap electrolyzer, it is essential to briefly discuss the methods used for quantifying 1,4-NADH. The unique hydrogen atom at position C_4 (**Figure 1B**) in the NADH structure generates a distinct signal in the ^{1}H-NMR spectrum, confirming the conversion of NAD^+ to NADH. However, this signal cannot distinguish between the 1,4-NADH monomer and its dimer.

While distinguishing between 1,4-NADH and other forms like 1,2-NADH and 1,6-NADH is possible, it presents some challenges. UV-Vis spectroscopy, for instance, can differentiate 1,4-NADH by its absorption band at 340 nm, but it overlaps with similar wavelengths absorbed by both 1,6-NADH and the dimers of 1,4-NADH and 1,6-NADH (**Figure S2**).[21] For accurate quantification of active 1,4-NADH, enzymatic assays remain the most reliable approach.[1,22] Although commercially available, these assays are highly dependent on the sample matrix's specific properties. For example, the pH of the sample can significantly affect the enzymatic

reaction, either inhibiting or promoting its efficiency, necessitating careful pH adjustment or the selection of an enzyme suited for the task.

Analytic spectra for the quantification of NADH

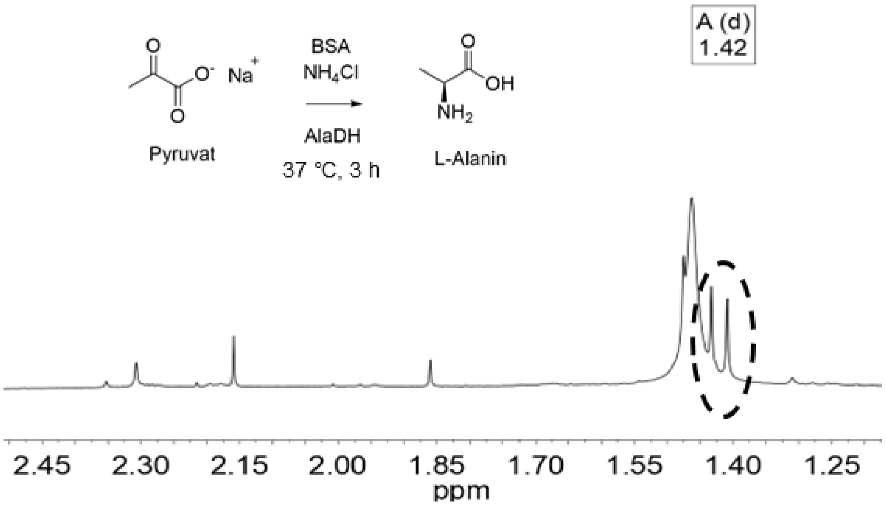

Figure S1. Exemplary ^{1}H-NMR spectrum of a product sample after conduction of the alanine dehydrogenase assay and the corresponding enzymatic reaction scheme (the marked doublet corresponds to the methyl group of alanine; its integral compared to that of an internal standard was used to determine the amount of present 1,4-NADH).

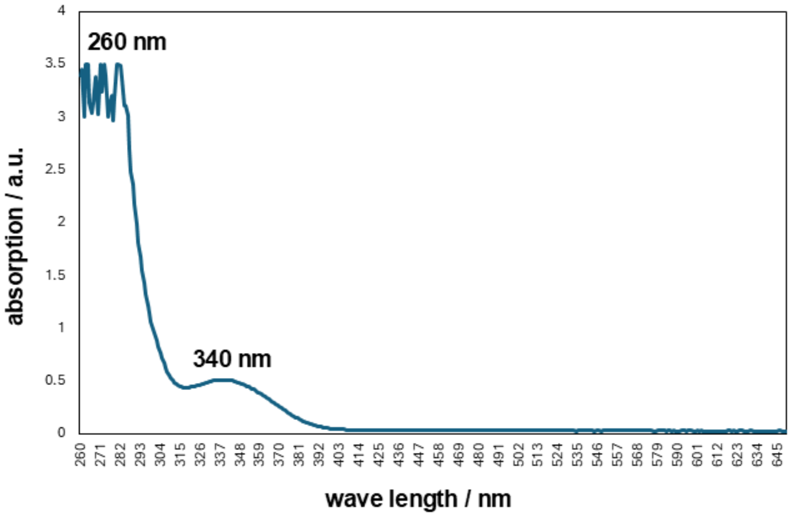

Figure S2. UV-Vis spectrum of an exemplary product sample from the direct electrochemical reduction of NAD^+ (absorption band at 260 nm corresponds to all NADH derivates; absorption band at 340 nm corresponds to 1,4-NADH and potentially to 1,6-NADH and the oligomers of both isomers).

Flow cell setup

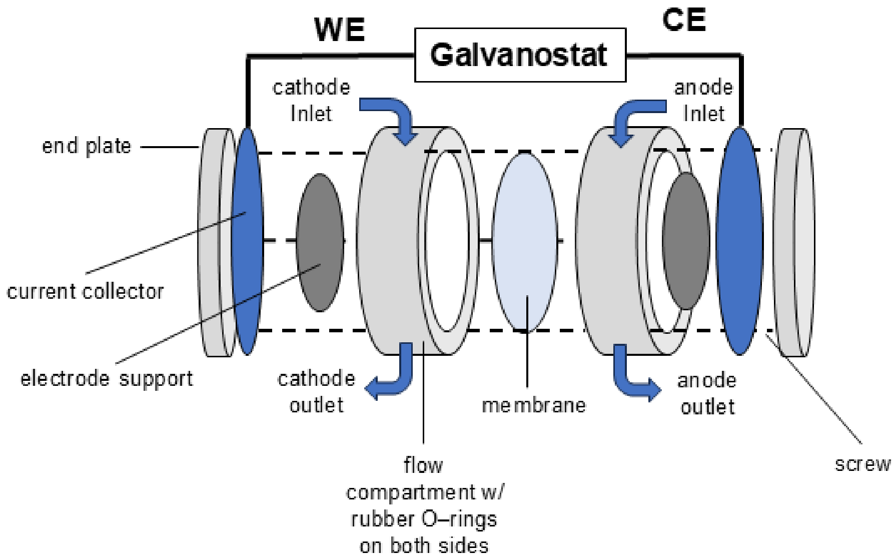

Figure S3. Continuous flow reactor configuration used for the direct electrochemical regeneration of NADH from NAD^{+} (the electrolytes were recirculated through the cell using a peristaltic piston pump).

Setup coupling electrochemical NADH regeneration with enzyme catalysis

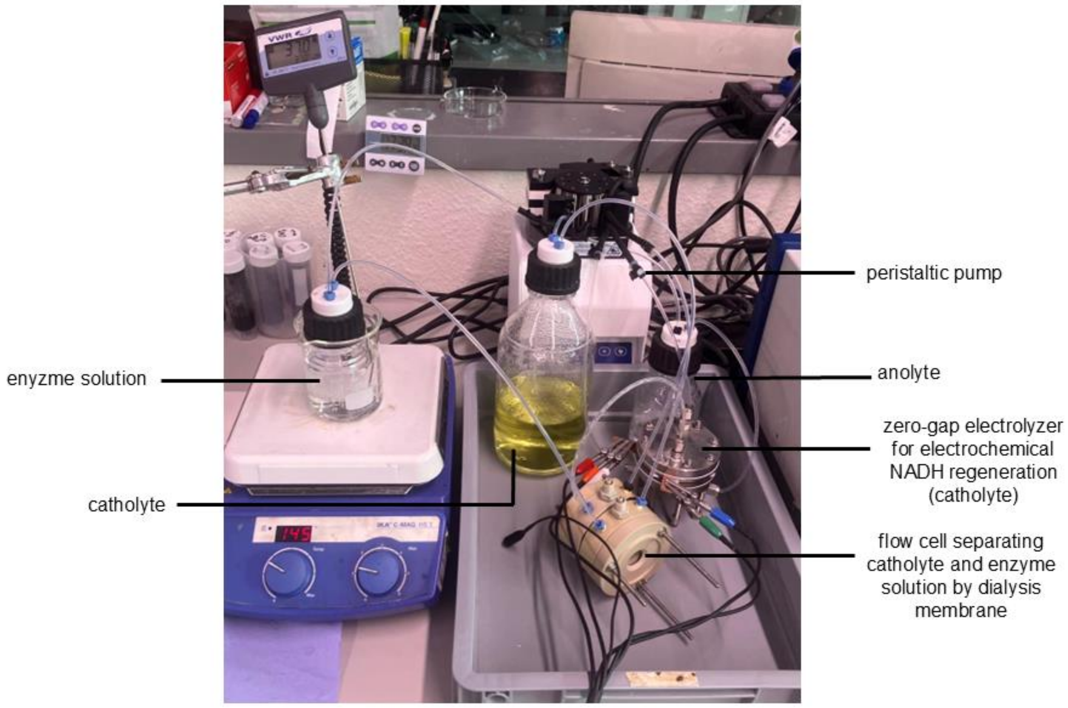

Figure S4. Image of the enzymatic conversion of acetophenone to 1-phenylethanol with an NADH-dependent alcohol dehydrogenase coupled with the electrochemical regeneration of NADH.

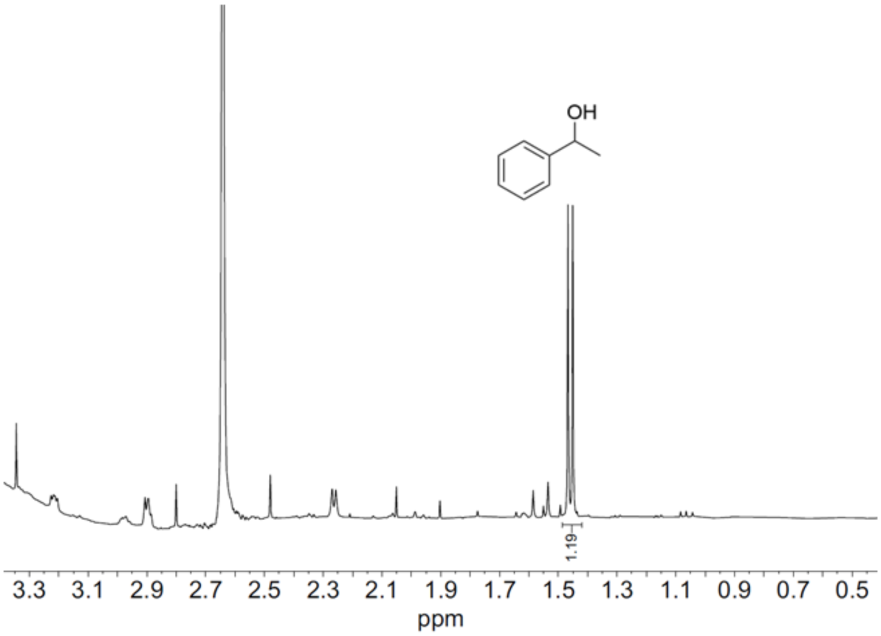

Figure S5. ^{1}H-NMR-spectrum of the enzyme solution after operation of the setup from **Figure S4** for 24 h at a current density of 10 mA cm^{-2}.

Compiled data of all conducted measurements

Table S1. Overview of the electrolysis experiments of NAD^+ in a zero-gap electrolyzer (EP: electrodeposited on carbon paper; NP: nanoparticles spray-coated onto carbon paper; UV: according to UV-Vis spectroscopy; RR: reaction rate; enzyme: according to the alanine dehydrogenase enzyme assay).

catalyst	current density (mA cm^{-2})	electrolyte volume (ml)	FE (UV) (%)	RR (UV) (µmol h^{-1})	RR (enzyme) (µmol h^{-1})	Selectivity (1,4-NADH) (%)
Cu_{mesh} (fine)	6	17	49	97	-	
Cu_{NP}	10	50	8	199	-	
Ag_{NP}	10	50	52	1216	148	12
Ag_{EP}	10	30	85	1120	-	
Cu_{EP}	10	30	9	119	-	
Cu_{mesh} (fine)	10	30	70	1635	-	
Cu_{mesh} (fine)	50	150	61	7116	-	
Cu_{mesh} (fine)	75	225	60	10489	148	1
Cu_{mesh} (fine)	100	300	29	6708	258	4
Cu_{mesh} (fine)	130	100	5	2955	144	5
Cu_{mesh} (fine)[a]	1	30	100	650	0	0
Cu_{mesh} (coarse)	100	275	11	1438	683	47
Cu_{mesh} (intermediate)	100	275	8	1056	792	75
Cu_{EP}	30	90	5	180	7	4
Cu_{EP}[a]	30	90	4	163	-	
Cu_{mesh} (fine)	30	90	16	597	-	
Cu_{mesh} (fine)[b]	30	90	15	650	-	
Ti_{felt}	100	300	21	790	459	58
Ti_{felt}	30	90	10	356	221	62
Ti_{felt}	10	92	13	178	194	100
Ti_{felt}	130	100	5	2570	174	7
Ti_{felt}[a]	1	30	100	470	0	0

[a]the measurements were carried out in a continuous flow electrolyzer with a membrane-electrode gap of 1 cm; FE (UV) were calculated to be above 100% due to statistical error

[b]the applied flow rate merely transported a third of the theoretically convertible amount of NAD^+ through the electrolyzer
